# Focando no Ventrículo Direito na Síndrome PRKAG2

**DOI:** 10.36660/abc.20220795

**Published:** 2022-11-23

**Affiliations:** 1 St Bartholomew’s Hospital Barts Heart Centre Londres Reino Unido Barts Heart Centre, St Bartholomew’s Hospital, Londres - Reino Unido; 2 University College London Institute of Cardiovascular Science Londres Reino Unido Institute of Cardiovascular Science, University College London, Londres - Reino Unido

**Keywords:** Síndrome PRKAG2, Ecocardiografia/métodos, Hipertrofia Ventricular Direita, Deformação Miocárdica, Volume Sistólico

O gene PRKAG2 codifica a subunidade reguladora gama 2 da proteína quinase ativada por monofosfato de adenosina (AMPK). A AMPK tem um papel central na homeostase da energia celular. A variação patogênica no PRKAG2 causa uma síndrome autossômica dominante composta por hipertrofia ventricular, arritmias supraventriculares, pré-excitação eletrocardiográfica e anormalidades do sistema de condução.^1-3^ A base genética desta apresentação sindrômica foi descoberta em 2001.^4^ Histologicamente há acúmulo de glicogênio no miócito, e o padrão típico de hipertrofia é geralmente descrito como biventricular e concêntrico, semelhante a outras fenocópias metabólicas da cardiomiopatia hipertrófica (CMH),^5^ com disfunção sistólica como outra possível característica “red-flag”. As variantes patogênicas mais frequentes são p.Arg302Gln e p.Asn488Ile.^1^ Devido à raridade da condição, a maioria das publicações consiste em pequenas séries de casos ou relatos de casos, com poucas exceções.^3^ Nenhuma das publicações anteriores relata especificamente os achados de imagem do ventrículo direito nessa condição.

Expandindo seu trabalho anterior, onde os achados de ecocardiografia 3D e imagem de strain do ventrículo esquerdo foram descritos em uma coorte de 30 pacientes com síndrome PRKAG2,^6^ Pena et al.,^7^ fornecem um breve relatório com foco no ventrículo direito (VD), usando a mesma coorte, na edição atual desta Revista.

Achados relevantes incluem uma alta prevalência de hipertrofia do VD (presente em 27 de 30 pacientes), uma redução mais significativa do strain basal do ventrículo direito em comparação com os segmentos médio e apical e uma fração de ejeção do VD anormalmente baixa em 17 pacientes (que é inferior a 35% em 7). É importante ressaltar que apenas 4 pacientes apresentaram aumento das pressões sistólicas da artéria pulmonar, portanto, não parece que as anormalidades do VD sejam secundárias ao aumento das pressões de enchimento à esquerda. Os achados esperados incluíam pacientes com marca-passo com pior fração de ejeção do VD e pior deformação miocárdica e uma correlação entre o strain ventricular direito e a fração de ejeção.

Esses achados confirmam a impressão de hipertrofia biventricular como característica dessa condição, em comum com outras doenças metabólicas.^5^ O estudo também mostrou alta prevalência de disfunção sistólica do VD, com consequências clínicas e prognósticas potencialmente significativas. Investigações anteriores mostraram que a hipertrofia do VD na CMH foi correlacionada com um escore de risco de morte súbita cardíaca calculado aumentado e independentemente relacionado à presença de arritmias ventriculares.^8^ O próximo passo relevante será a investigação do impacto clínico desse envolvimento do VD em termos de desfechos na síndrome PRKAG2.
