# Supplementary material for: Focando no Ventrículo Direito na Síndrome PRKAG2
Source: Arq Bras Cardiol. 2022 Nov 23;119(6):910–1. [Article in Portuguese] doi: 10.36660/abc.20220795 (PMC9814802; doi:10.36660/abc.20220795)
Supplement: Translation

# Translation: Focusing on the Right Ventricle in PRKAG2 Syndrome

**Affiliations:** 1 London United Kingdom Barts Heart Centre, St Bartholomew’s Hospital, London - United Kingdom; 2 London United Kingdom Institute of Cardiovascular Science, University College London, London - United Kingdom

**Keywords:** PRKAG2 Syndrome, Echocardiography/methods, Hypertrophy, Right Ventricle, Myocardial Deformability, Stroke Volume

The *PRKAG2* gene encodes the adenosine monophosphate-activated protein kinase (AMPK) gamma 2 regulatory subunit. AMPK has a central role in cellular energy homeostasis. Pathogenic variation in *PRKAG2* causes an autosomal dominant syndrome comprising ventricular hypertrophy, supraventricular arrhythmias, electrocardiographic pre-excitation and conduction system abnormalities.^1-3^ The genetic background of this syndromic presentation was discovered in 2001.^4^. Histologically there is myocyte glycogen accumulation, and the typical pattern of hypertrophy is usually described as biventricular and concentric, similar to other metabolic phenocopies of hypertrophic cardiomyopathy (HCM),^5^ with systolic dysfunction as another possible “red-flag” feature. The more frequent pathogenic variants are p.Arg302Gln and p.Asn488Ile.^1^ Due to the condition’s rarity, most publications consist of small case series or case reports, with few exceptions.^3^. None of the previous publications specifically report right ventricular imaging findings in this condition.

Expanding on their previous work, where 3D echocardiography and strain imaging findings of the left ventricle were described in a cohort of 30 patients with PRKAG2 syndrome,^6^ Pena et al.^7^ provide a short report focusing on the right ventricle (RV), using the same cohort, in the current edition of this Journal.

Relevant findings include a high prevalence of RV hypertrophy (present in 27 out of 30 patients), a more significant basal right ventricular stain reduction compared to mid and apical segments, and an abnormally low RV ejection fraction in 17 patients (which is below 35% in 7). Importantly, only 4 patients had increased pulmonary artery systolic pressures, so it does not seem that the RV abnormalities are secondary to increased filling pressures from the left. Expected findings included patients with pacemakers having worse RV ejection fraction and poorer myocardial deformation and a correlation between right ventricular strain and ejection fraction.

These findings confirm the impression of biventricular hypertrophy as a characteristic feature of this condition, in common with other metabolic diseases.^5^ The study also showed a high prevalence of RV systolic dysfunction, with potentially significant clinical and prognostic consequences. Previous investigations have shown that RV hypertrophy in HCM was correlated with an increased calculated sudden cardiac death risk score and independently related to the presence of ventricular arrhythmias.^8^ The relevant next step will be an investigation of the clinical impact of this RV involvement in terms of outcomes in the PRKAG2 syndrome.
